# Oncogenic microRNA 17-92 cluster is regulated by epithelial cell adhesion molecule and could be a potential therapeutic target in retinoblastoma

**Published:** 2012-08-28

**Authors:** Moutushy Mitra Kandalam, Madhu Beta, Uma K. Maheswari, S. Swaminathan, Subramanian Krishnakumar

**Affiliations:** 1 Department of Ocular Pathology, Vision Research Foundation, Sankara Nethralaya, Chennai, Tamil Nadu, India; 2 Centre for Nanotechnology and Advanced Biomaterials, Shanmugha Arts, Science, Technology and Research Academy University, Tanjore, India

## Abstract

**Purpose:**

Several miRNAs have been reported as candidate oncogenes and tumor suppressors, which are involved in the pathways specifically altered during tumorigenesis or metastasis. The miR 17–92 cluster located in 13q31 locus might contribute to retinoblastoma (RB) oncogenesis as 13q31 is amplified often in RB. We attempted to identify the factors involved in the regulation of miR 17–92 cluster in RB.

**Methods:**

Real-time quantitative reverse transcriptase PCR was performed to study the expression of the miR 17–92 cluster in primary RB tumors and in Y79 cells after epithelial cell adhesion molecule (EpCAM) silencing. EpCAM was silenced using siRNA and confirmed by western blotting. The Y79 cells were transfected with individual and mixed antagomirs and studied the cell viability by (3-(4,5-Dimethylthiazol-2-yl)-2,5-diphenyltetrazolium bromide (MTT) assay, invasion by matrigel analysis and caspase-3 expression by flow cytometry.

**Results:**

The relative expression of miR 17–92 cluster, compared to that of a normal retina, ranged from 25 to 220 fold (p<0.0001), miR-18 being highly expressed in RB. Post EpCAM silencing resulted in a significant decrease (p<0.01) in the expression of the miR 17–92 cluster by 4 to eightfold in Y79 cells. Y79 cells transfected with an antagomirs mix (all 5 miRNAs) showed decreased cell viability (p<0.001) and cell invasion (p<0.001). Similarly, Y79 cells treated with antagomirs mix showed increased expression of caspase-3 (p<0.001), which confirms the anti-proliferative effect of antagomirs.

**Conclusions:**

This study has showed varied expression of the miR17–92 cluster in primary RB tumors. EpCAM influences miR 17–92 cluster expression in retinoblastoma. In addition, we showed that the miR 17–92 cluster plays a role in RB cell proliferation and invasion. Therefore, targeting the miRNA 17–92 cluster may be beneficial for controlling Y79/RB cell proliferation and invasion.

## Introduction

MicroRNAs (miRNAs) are small (18–25 nt) non-coding RNAs that play an important role in regulating a variety of biologic processes by silencing specific target genes [1]. Although humans contain only about 800–1000 miRNAs [2,3], it is believed that these small RNAs are able to control a major portion (more than 30%) of all protein-coding genes [4]. Many research reports have revealed that miRNAs participate in the control of numerous biologic processes, such as cell differentiation, proliferation and apoptosis, development, immunity, metabolism and stem cell maintenance [5-12]. Several miRNAs have been reported as candidate oncogenes and tumor suppressors, which are involved in the pathways specifically altered during tumorigenesis or metastasis [13-16].

The potential oncogenic microRNA 17–92 cluster is our interest in the present study because this cluster is located at 13q31.3, which lies near the minimal common region of gain (MRG) in retinoblastoma [17]. The more common chromosomal gains and losses in retinoblastoma (RB) have attracted the most research attention and have yielded genes of general importance in cancer. Regions showing occasional gains are particularly intriguing for further research, as they may point to oncogene candidates.

Previous reports based on comparative genomic hybridization (CGH) show that 13q is often gained in RB tumors [18]. Therefore, we hypothesized that dysregulation of this miR 17–92 oncomir cluster might contribute to RB oncogenesis. However, a recent report published by Karina et al. [19] states that high expression of the miR 17–92 cluster did not correlate to the genomic amplification of miR 17–92 locus (13q31) in RB tumors. Therefore, further studies are needed to understand the genes or proteins involved in the regulation of the miR 17–92 cluster in retinoblastoma. Schulte et al. [20] have demonstrated that miR-106a and miR-17 clusters, which have previously been shown to be regulated by c-Myc, were also induced by myelocytomatosis viral related oncogene, neuroblastoma (MYCN) overexpression in neuroblastoma. With regards to MYCN’s role in retinoblastoma, we recently showed through expression microarray studies that when epithelial cell adhesion molecule (EpCAM) protein was knocked down, MYCN protein was down-regulated in RB cells, providing evidence that MYCN expression is regulated by EpCAM in retinoblastoma [21]. In this milieu, we hypothesize that EpCAM may also be involved in regulating the miR 17–92 cluster in retinoblastoma. In the present study, we attempted to determine the relationship between EpCAM and the miR 17–92 cluster in retinoblastoma. We also provide the relative quantification levels of the miR 17–92 cluster in a large cohort of primary RB tumors compared to normal retinal tissues. In addition, we demonstrate the role of individual miRNA from the miR 17–92 cluster in RB cell proliferation and invasion.

In the present study, we studied the expression of miR 17–92 in a reasonably large cohort of primary RB tumors and found that miR 17–92 cluster is overexpressed in the RB primary tumors compared to non-neoplastic retina. The cell proliferation and invasion potential of the Y79 RB cell line was significantly decreased in response to knocking down the miR 17–92 cluster. Significantly, inhibition of EpCAM led to a reduction in the expression of this miR 17–92 cluster and also showed a reduction in cell proliferation and cell invasion ability. To the best of our knowledge, these results provide the first evidence that the miR 17–92 cluster may functionally contribute to RB tumor progression and also reveal a possible novel regulatory link between the miR 17–92 cluster and human EpCAM-expressing RB tumors.

## Methods

All samples were collected with the approval of the institutional review board at VRF (Vision Research Foundation) and in accordance with the Declaration of Helsinki. This study was conducted at the Medical Research Foundation and Vision Research Foundation, Sankara Nethralaya, India, and was approved by the Vision Research Foundation ethics board.

### MicroRNA extraction

Micro RNA was extracted with a mirVana ^TM^ miRNA extraction kit (Ambion, Austin, TX) according to the manufacturer’s instructions. In brief, fresh RB tissues were weighed and lysed using 10 volumes of Lysis/Binding Buffer per tissue mass into a tube set on ice and homogenized. One-tenth volume of miRNA homogenate additive was added to the cells and tissue lysate (or homogenate), and mixed well by vortexing or inverting the tube several times and keeping the mixture on ice for 10 min. Acid-Phenol: Chloroform was added to a volume that is equal to the lysate volume before the addition of the miRNA homogenate additive and vortexed for 30–60 s to mix. The mixture was centrifuged for 5 min at maximum speed (10,000× g) at room temperature to separate the aqueous and organic phases. After careful removal, the aqueous (upper) phase was transferred to a fresh tube and 1.25 volumes of room temperature, 100% ethanol was added to the aqueous phase. For each sample, a filter cartridge was placed into one of the collection tubes and the lysate/ethanol mixture (from the previous step) was added onto the filter cartridge. Centrifugation was done for ~15 s at 10,000× g to pass the mixture through the filter. Flow-through was discarded and the step was repeated until all of the lysate/ethanol mixture was passed through the filter. In the next step, 700 μl miRNA wash solution 1 (working solution mixed with ethanol) was added to the filter cartridge and centrifuge for ~5–10 s to pull the solution through the filter. Next, 500 μl Wash Solution 2/3 (working solution mixed with ethanol) was applied and passed through the filter cartridge as in the previous step, which was repeated with a second 500 μl aliquot of Wash Solution 2/3. After discarding the flow-through from the last wash, a preheated elution (95 °C) solution was added to the center of the filter. The cap was closed and centrifuged for ~20–30 s at maximum speed to recover the RNA. The eluate (which contains the RNA) was stored at –80 °C until further use.

### Real-time Quantitative Reverse transcription PCR

The detection and quantification of mature miRNA was achieved using reverse transcription– real-time PCR. All reagents, including the TaqMan® MicroRNA Individual Assays miR-17 (assay ID, 002308), hsa-miR-19b-1 (assay ID, 002425), hsa-miR-20a (assay ID, 000580), hsa-miR-18a (assay ID, 002422), hsa-miR-92 (assay ID, 002137), the TaqMan® MicroRNA Reverse Transcription Kit and the TaqMan® Universal PCR Master Mix without AmpErase® UNG, were purchased from Applied Biosystems (Foster City, CA). Quantification was performed using the manufacturer’s protocol starting with 10 ng of the total RNA sample. As a control for normalization, the U6 miRNA was used. The PCR products were detected with an ABI PRISM 7500 sequence detection system and analyzed with the ABI PRISM 7500 SDS software (Applied Biosystems). The cycle threshold value (C_T_) was determined for each miRNA and the relative amount of each miRNA to U6 was calculated using the expression – 2^-ΔCT^, where ΔC_T_=(C_T_miRNAtest – C_T_ control miRNA). The mean relative miRNA expression±SEM in tumor samples compared to normal tissue was calculated and expressed as a ratio. The data were subjected to a Student’s paired *t*-test, and p<0.05 was taken to indicate a significant difference. Tumors with a greater than twofold increase in the expression of each miRNA were also noted. Gene expression assays for *EpCAM* (Hs00158980_m1) and glyceraldehyde-3-phosphate dehydrogenase (*GAPDH*; Hs99999905_ml), were obtained from Applied Biosystems (LabIndia, Chennai, India).

### Transfection of AntagomiRs in Y79 retinoblastoma cells

Y79 cells were seeded in an antibiotic-free medium at 50%–60% confluence (6x10^5^ /well cells in six well plate) and transfected with individual antagomirs (micro RNA power inhibitors; hsa-miR-17 miRCURY LNA- 426848–00; hsa-miR-19b miRCURY LNA-426922; hsa-miR-18a miRCURY LNA-426873; hsa-miR-92a miRCURY LNA-427466; hsa-miR-20a miRCURY LNA-426943; and Negative Control A miRCURY LNA- 199020–08, purchased from Exiqon, Vedbaek, Denmark) each at a final concentration of 2.5 µM or with an equal volume of PBS. Cells were stained with trypan blue and viable cells were counted 1, 2, and 4 days after transfection. Cells were harvested 48 h and four days after transfection for proliferation/invasion and real-time PCR analysis, respectively.

### In vitro cell proliferation assay

For the evaluation of cell proliferation rate, cells were seeded on 96-well plates at a density of 1x10^4^ cells/well in the complete RPMI medium, followed by the methyl thiazol tetrazolium assay (MTT assay, Sigma-Aldrich, Bangalore, India). The amount of MTT formazan product was determined using a microplate reader and an absorbance of 560 nm (Beckman Coulter, Nyon, Switzerland).

### In vitro cell invasion analysis

The 24-well plate Transwell system with a polycarbonate filter membrane of 8-mm pore size (Corning, New York, NY) was used. The cell suspensions were seeded in the upper compartment of the Transwell chamber at the cell density of 1×10^5^ in 100 µl serum-free medium. After 24 h, the medium was removed and the filter membrane was fixed with 4% formalin for 1 h. The opposite surface of the filter membrane, which faced the lower chamber, was stained with methylene blue for 3 min and the migrated cells were then visualized under an inverted microscope.

### RNA interference

Gene silencing of the EpCAM expression was performed essentially as described previously using sequence-specific siRNA and transfection reagents [22]. Briefly, 1×10^5^ cells were plated in each well of the six-well plates and allowed to grow for 24–36 h (until they were 40%–60% confluent). siRNA was then transfected into cells at a concentration of 200 nM using Hi-Perfect transfection reagent (Qiagen, Santa Clara, CA) and a serum-free medium. After 4 h of incubation, a serum-rich medium was added. Human *EpCAM* siRNA (Hs_TACSTD1_10; catalog number SI04343416; Forward strand: GGA ACU CAA UGC AUA ACU ATT; and the reverse strand: UAG UUA UGC AUU GAG UUC CCT) and scrambled siRNA (catalog number 1022563) were used in this study.

### Western blotting

Cells were lysed in radioimmunoprecipitation assay lysis buffer for 15 min on ice. An aliquot (100 µg) of lysate was electrophoresed with 10% sodium dodecyl sulfate-polyacrylamide gel and blotted onto a nitrocellulose membrane. Membranes were blocked in 5% fat-free milk and then incubated separately with 1:500 diluted mouse monoclonal primary antibody against EpCAM (C-10) overnight at 4 °C. β-actin was used as a loading control (AC-15, dilution: 1:4,000; Sigma). After washing, the membranes were incubated with horseradish peroxidase-conjugated antimouse gamma immunoglobulin (IgG) antibody (diluted to 1:2,000; Santa Cruz Biotechnology, Santa Cruz, CA) for 1 h at room temperature. The bands were visualized using an enhanced chemiluminescence kit (Amersham, Pittsburgh, PA). Each experiment was performed in triplicate.

### Caspase-3 assay by flow cytometer

For intracellular staining, cells were fixed and permeabilized with 2% paraformaldehyde and 0.05% Tween-20 to allow intracellular labeling with the cleaved caspase 3 antibody (1: 200; Cell Signaling) was added and kept for 1 h incubation at 4 °C. Following incubation, the cells were washed twice with ice-cold PBS and incubated with secondary FITC -anti rabbit- IgG (1:1,000 dilution; Sigma) for 30 min at 4 °C. The cells were then washed twice with ice-cold PBS and resuspended in an FACS buffer and analyzed with an FACS Calibur (BD Biosciences, Heidelberg, Germany).

### Statistical analysis

All experiments were repeated at least three times. Independent *t*-test analysis and the Pearson correlation test (Pearson correlation; v1.0.3) were used to conduct statistical analysis. The data are presented as means±standard deviation (SD). The differences were considered significant for p values of <0.05.

## Results

### Clinical profile

The clinical features of the 19 cases are summarized in Table 1. The mean age of patients included in the study was 29.5±21.8 months (range 5 mon to 9 years) and the group comprised 13 boys and 6 girls. All the tumors were sporadic cases, with right eye involvement in ten cases and left eye in nine cases. The summary of clinical and histologic findings is provided in Table 1.

**Table 1:** Clinicophenotypical Correlation of markers in nineteen Cases of RB and expression fold change of miR 17–92 clusters in each tumor relative to normal retina as quantified by QPCR.

**Serial No**	**Age/sex**	**Clinicopathological features**	**miR-17**	**miR-18a**	**miR-19b**	**miR-20a**	**miR-92**
1	2 y/M	OS,unilateral, PD, prelaminar & post laminar invasion of ON, scleral invasion	25	120	13	36	45
2	1 y/M	OD, unilateral,WD, prelaminar & laminar invasion of ON	15	206	4	38	70
3	3 y/F	OS, unilateral,MD, prelaminar and post laminar ON invasion	45	290	19	25	26
4	2 y/M	OD, PD, focal choroidal invasion < 3 mm	65	225	36	34	45
5	2 y/F	OD, PD, minimal prelaminar invasion of ON	43	70	11	22	2
6	4 y/M	OD, unilateral,MD, prelaminar & post laminar invasion of ON	40	201	32	38	72
7	7 y/F	OS, unilateral,PD, No invasion of choroid and ON	35	156	17	32	38
8	3 y/M	OS, unilateral,PD, No invasion of choroid and ON	75	368	29	50	38
9	8 y/F	OS, unilateral,PD, focal choroidal invasion <3 mm	44	78	32	41	32
10	9 y/F	OD, unilateral, PD, post laminar invasion of ON.	68	301	15	65	71
11	2 y/M	OS, unilateral,PD, prelaminar & post laminar invasion of ON, scleral invasion	26	299	19	32	4
12	1 y/F	OS, unilateral,WD, prelaminar invasion of ON	65	167	26	15	32
13	2 y/M	OS, unilateral,PD, focal choroidal invasion <3 mm	51	100	39	22	15
14	5 Mon/M	OD, unilateral,PD, diffuse choroidal invasion > 3 mm & pre laminar/laminar ON invasion	40	179	36	40	2
15	4 y/M	OD, unilateral,PD, focal RPE and focal choroidal invasion < 3 mm.	20	255	24	45	68
16	2 y/M	OS, bilateral, PD, focal choroidal and pre laminar ON invasion	46	370	38	52	17
17	7 1/2 Mon/M	OD, unilateral, WD, focal choroidal invasion < 3 mm with pre and post laminar ON invasion.	49	320	29	49	28
18	32 Mon/M	OD, unilateral,PD, no invasion of choroid and ON	34	300	24	59	43
19	6 y/M	OD, unilateral,MD, full thickness choroidal invasion > 3mm	69	175	34	64	11

**Abbreviations:** M: Male; F: Female; Mon: months; Y: years; OD: right eye; OS: left eye; ON: optic nerve; PD: poorly differentiated; MD: moderately differentiated; WD: well differentiated.

### Relative Quantification of EpCAM, miRNA 17–92 cluster expression levels in primary tumor and control samples

**Figure 1:**
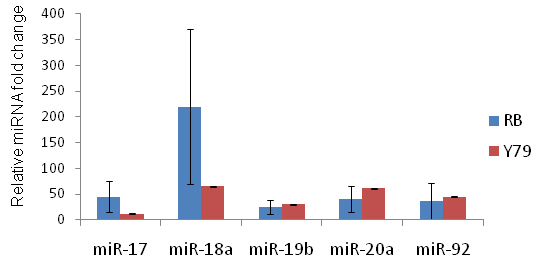
A graph showing the expression of miR 17–92 cluster in primary retinoblastoma (RB) tumors. The blue bars represent RB tumors and the red bars represent Y79 cells. The error bars represent the varying expression of microRNA in 19 tumors.

**Figure 2:**
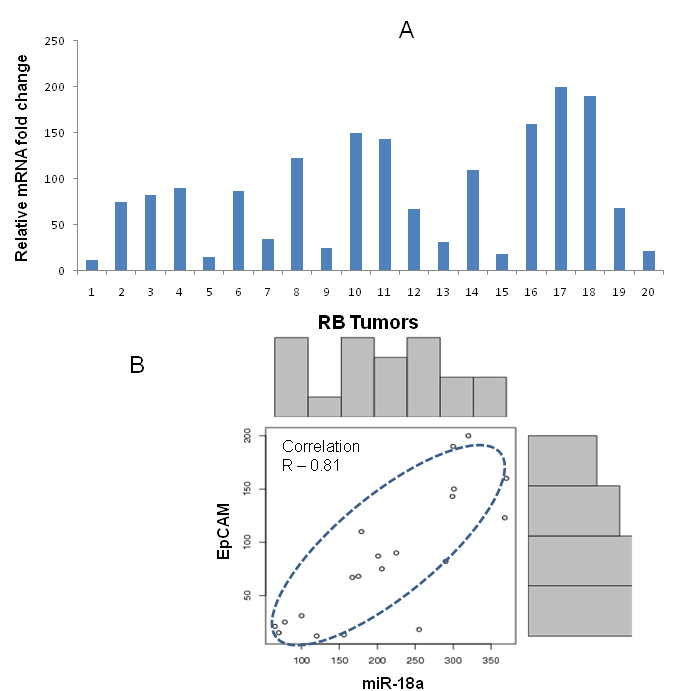
Correlation between miR-18a and EpCAM expression in RB tumors. **A**: A bar diagram showing the expression of EpCAM in retinoblastoma (RB) tumor samples. The expression ranged from 12-fold to 200 fold compared to normal control retina samples. **B**: The plot showing the correlation between EpCAM and miR-18a in primary RB tumors. The correlation R value is equal to 0.81 (strong correlation).

We used relative quantification method to determine the expression of miRNA 17–92 in 19 tumors samples (Figure 1) compared to three age-matched (13–18 months old) and three adult retinas (21–60 years). The miR-17–92 expression was also determined in Y79 cells. Levels were normalized to *U6* miRNA. The average fold change of the miRNA expressions in 19 primary RB tumors was plotted in the graph comparing them with the control samples (Figure 1). The relative expression of the miR 17–92 cluster, compared to that of the normal retina, ranged from 25 to 220 fold, with miR-18 being highly expressed (220 fold) in RB tumors (Table 1). The Y79 cell line showed 12 to 65 fold higher expression of the miR 17–92 cluster when compared to the normal retina. There was a statistically significant overexpression of the miRNA 17–92 clusters expression in tumors compared to age-matched and adult control retinas (p<0.0001). We also quantified the relative expression of EpCAM in 19 RB primary tumors. All the tumors expressed high levels of EpCAM when compared to normal control retinal samples. The expression of EpCAM ranged from 12-fold to 200 fold in RB tumors (Figure 2A). We did a Pearson correlation analysis to determine whether the expression correlated between EpCAM and individual miRNA. We found that there is a significant correlation (R=0.81) between the expression levels of EpCAM and miRNA-18 in the sample cohort (Figure 2B). However, we did not observe a statistically significant correlation with other miRNAs.

### EpCAM protein influences miR 17–92 expression in Y79 cells

**Figure 3:**
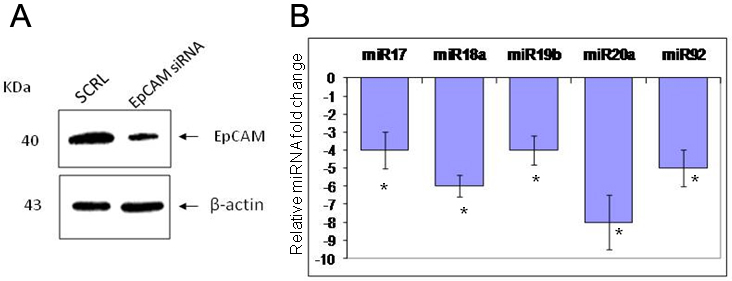
siRNA mediated EpCAM downregulation and its effect on miR 17–92 expression. **A**: The western blot shows the down-regulation of EpCAM in Y79 cells treated with EpCAM specific siRNA. The lower panel shows the Beta actin expression as loading controls. **B**: QPCR shows the change in the expression of the miR 17–92 cluster in Y79 cells treated with EpCAM siRNA. There is a significant down-regulation of the miR 17–92 cluster by 4 to eightfold (*p<0.01).

We studied the expression of miR 17–92 cluster with real-time quantitative PCR in Y79 cells after siRNA mediated silencing of EpCAM. We observed 50% down-regulation of EpCAM protein by western blotting analysis (Figure 3A). We observed that the miR 17–92 cluster expression was decreased after EpCAM silencing (Figure 3B). The down-regulation of all miR 17–92 ranged from fourfold to eightfold compared to the control Y79 cells (scrambled and siRNA treated). This indirectly shows that EpCAM influences the expression levels of miR 17–92 cluster in RB cells. After 72 h of EpCAM siRNA treatment, when the Y79 cells were passaged further, the miR 17–92 cluster expression was restored (data not shown).

### Functional role of miR 17–92 cluster in retinoblastoma

#### Effect of antagomirs on miR 17–92 expression

**Figure 4:**
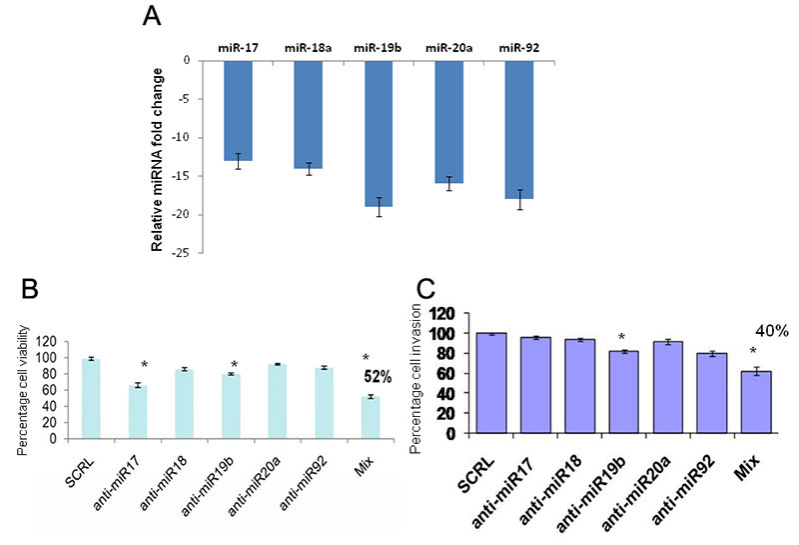
Effect of antagomirs on miR 17–92 cluster, cell viability and cellular invasion. **A**: The bar diagram shows the expression of miR 17–92 cluster in Y79 cells treated with specific antagomirs. There is a significant down-regulation of miR 17–92 clusters when treated with antagomirs. **B**: MTT assay graph showing the Y79 cell viability after 48 h of treatment with individual and mix antagomirs. There is a significant decrease in cell viability when treated with antagomirs-17 (*p<0.01), antagomirs-19b (*p<0.01) and antagomirs mix (*p<0.001). **C:** shows the effect of antagomirs (17–92) on Y79 cellular invasion process as analyzed by Matrigel invasion assay. There is a significant decrease in cell invasion when treated with antagomirs-19b (*p<0.05) and antagomirs mix (**p<0.001)

We observed significant down-regulation of the miR 17–92 cluster in Y79 cells treated with specific antagomirs (Figure 4A). The down-regulation of the miR 17–92 cluster ranged from 13 to 19-fold relative to the untreated Y79 cells.

#### Proliferation

The miR 17–92 cluster expression was inhibited using antagomirs specific to each individual miRNA. MTT assay was performed on Y79 cells after 48 h of treatment with antagomirs. The MTT assay shows a significant decrease in the cell viability of Y79 cells when treated with antagomirs-17 and anti-miR-19b. In addition, there was a significant decrease in cell viability (p<0.01) when they were treated with a mixture of all five antagomirs (anti-17–92; Figure 4B). This data clearly shows that the miRNA-17–92 cluster plays a role in Y79 cell proliferation.

#### Invasion

A matrigel invasion assay was performed on the Y79 cells after 48 h of treatment with antagomirs. There was a 20% decrease in Y79 cellular invasion when they were treated with antagomir-19b and there was a significant decrease in cell invasion (40%) (p<0.001) when they were treated with a mixture of all five antagomirs (anti-17–92; Figure 4C). Therefore, the miR 17–92 cluster may be involved in the Y79 cell invasive property. Targeting the miRNA 17–92 cluster may be beneficial for controlling Y79/RB cell proliferation and invasion processes.

### Caspase-3 expression in Y79 cells treated with antagomirs mix

Y79 cells treated with antagomirs mix (17, 18a, 19b, 20a, 92b) showed higher caspase-3 expression (45%) compared to control Y79 cells treated with scrambled antagomirs. The increase in caspase-3 expression reflects the apoptotic events in Y79 cells treated with antagomirs. This confirms that the miR 17–92 cluster inhibition may be beneficial in controlling RB tumor cell proliferation.

## Discussion

In the present study, we quantified miRNA 17–92 clusters in 19 tumors samples (Figure 1). For controls, miRNA 17–92 cluster levels were determined in 3 age-matched (13–18 months old), 3 adult retinas (21–60 years), and 1 RB cell line (Y79). Levels were normalized to U6 miRNA. The average fold change of the miRNA expression in 19 primary RB tumors was plotted in the graph comparing them against the control samples. (Figure 1). There was a statistically significant overexpression of miRNA 17–92 clusters expression in tumors compared to normal control retinas (p<0.0001). Our results demonstrate that the expression levels of miR 17–92 clusters vary widely within the primary RB tumors. Karina et al. previously demonstrated that a high expression of miR 17–92 cluster is not related to the genomic amplification of miR 17–92 locus [19] in RB tumors. Therefore, Karina et al. [19] concluded that the high expression of miR 17–92 cluster might confer a selective advantage upon cells that have the potential to form retinoblastoma. However, further studies are needed to clearly explain the wide range of miR 17–92 expression levels in RB tumors.

In the present study, we attempted to understand the relationship between the EpCAM and miR 17–92 cluster. Therefore, we conducted an in vitro study and observed that miR 17–92 cluster expression went down when the EpCAM gene was silenced using EpCAM-specific siRNA. This study indirectly confirms that EpCAM protein regulates the expression of the miR 17–92 cluster in an RB cell line. This mechanism is possible because the intracellular domain of the EpCAM (ICD) has been shown to be involved in the transcription of certain genes [23]. In relation to this, we earlier demonstrated that EpCAM silencing decreases the expression of the *MYCN* gene in RB [21]. This is the first study to show the relationship between the EpCAM and miR 17–92 cluster in retinoblastoma.

**Figure 5:**
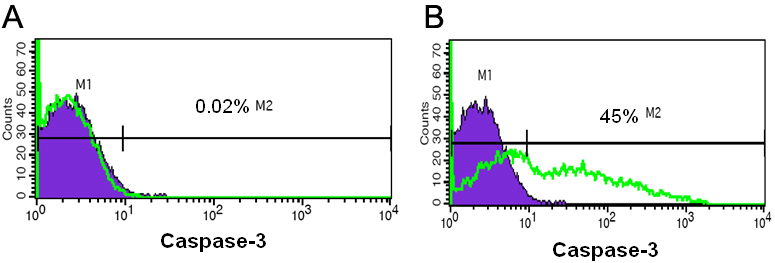
Transfection of antagomir mix induces caspase-3 expression in RB cells. **A**: Shows the flow cytometry density plot for caspase-3 expression in Y79 cells treated with control antagomir. The percentage of caspase-3 is negligible (less than 1%). **B**: Shows the flow cytometry density plot for caspase-3 expression in Y79 cells treated with antagomirs mix. There is a significant increase in caspase-3 expression (32%) in Y79 cells treated with antagomirs mix (*p<0.001).

Later, we attempted to investigate the individual miRNA role in retinoblastoma cells in vitro. The Y79 cells were transfected with individual antagomirs to miR-17, 18a, 19b, 20a, and 92, and studied the cell viability and cellular invasion process. We observed that there was a significant decrease in cell viability of Y79 cells when treated with antagomirs-17 (p<0.01) and anti-miR-19b (p<0.01) but not significantly when treated individually with 18a, 20a, and 92b. However, there was a significant decrease in cell viability (p<0.01) when treated with mixture of all five antagomirs (anti-17–92; Figure 3A). This data clearly shows that miRNA-17–92 cluster plays a role in Y79 cell proliferation. To support this, we performed a flow cytometry analysis to show the caspase-3 expression as evidence for apoptotic cascade on Y79 cells and found higher caspase-3 expression in Y79 cells treated with antagomirs mix (Figure 5) compared to scrambled miRNA treated Y79 cells. Subsequently, we investigated the role of miR 17–92 cluster in Y79 cell invasion by subjecting the antagomirs transfected Y79 cells to Matrigel invasion assay. We observed that there was 20% decrease in Y79 cellular invasion when treated with antagomir-19b (p<0.05) and there was significant decrease in cell invasion (40%; p<0.001) when treated with mixture of all five antagomirs (anti-17–92; Figure 3B). Therefore, miRNA 17–92 cluster may be involved in Y79 cell invasive property. Earlier, reports have shown that miR 17–92 cluster inhibits PTEN expression in human hepatocellular cancer and mouse lymphoproliferative disorders, and resulted in increased tumor cell proliferation, migration, and invasion [24-26].

To conclude, this study shows the varied expression of miR17–92 cluster in primary RB tumors. EpCAM influences miR 17–92 cluster expression in retinoblastoma. In addition, we showed that miR 17–92 cluster plays a role in RB cell proliferation and invasion. Therefore, targeting the miRNA 17–92 cluster may be beneficial for controlling Y79/RB cell proliferation and invasion.
